# Transcatheter aortic valve replacement for aortic regurgitation secondary to aortic sinus dilation with incidentally detected aortic chordae tendineae: case report

**DOI:** 10.3389/fcvm.2025.1711611

**Published:** 2025-11-14

**Authors:** Lingli Li, Lulu Liu, Wenjuan Bai

**Affiliations:** Department of Cardiology, West China Hospital, Sichuan University, Chengdu, China

**Keywords:** aortic chordae tendineae, transcatheter aortic valve replacement, aortic regurgitation, aortic sinus dilation, transesophageal echocardiography

## Abstract

**Introduction:**

Aortic chordae tendineae (ACT) are rare fibrous strands originating from early aortic valve cusp formation as embryonic remnants. Most aortic chordae tendineae are asymptomatic, but they may cause aortic regurgitation via chordal rupture or cusp restriction. This case presents transcatheter aortic valve replacement (TAVR) for aortic regurgitation secondary to aortic sinus dilation with incidentally detected ACT that did not directly contribute to valvular dysfunction.

**Patient presentation:**

An 81-year-old female with hypertension presented with progressive dyspnea. Cardiac auscultation revealed a diastolic murmur over the left sternal border. Transthoracic echocardiography demonstrated severe aortic regurgitation due to significant aortic root dilatation. Transapical TAVR was planned. Prosthetic size was determined via computed tomography. Pre-procedure transesophageal echocardiography (TEE) identified two ACTs connecting the free margin of the non-coronary cusp to the aortic sinus wall. TAVR with a self-expanding valve was performed, with intraoperative TEE confirming stable deployment despite rupture of two ACTs. The patient recovered uneventfully without perioperative complications, and follow-up transthoracic echocardiography confirmed optimal valve position with no paravalvular leakage.

**Conclusion:**

ACT presence does not preclude TAVR but requires meticulous intraoperative surveillance, TEE is critical for detecting these structures and guiding TAVR planning. Precise intraoperative positioning of valve graspers ensures avoidance of ACT-related complications, and rupture of ACT confirmed that it would not compromise prosthetic valve stability in the future. This case supports the feasibility of TAVR in complex aortic root anatomies with incidentally detected ACT.

## Introduction

Aortic chordae tendineae (ACT) are rare fibrous strands originating from early aortic valve cusp formation as embryonic remnants. Current case reports link ACT-induced regurgitation/stenosis predominantly to middle-aged to elderly patients (1). This suggests most ACT remain clinically silent unless valvular dysfunction arises from age-related cardiac changes, functional imbalance, or spontaneous chordal rupture. With the growing application of transcatheter aortic valve replacement (TAVR) (2), questions persist regarding ACT's potential influence on valve deployment and optimal procedural strategies. This case uniquely presents TAVR management for aortic regurgitation (AR) in the presence of incidentally detected ACT, highlighting intraoperative considerations and clinical implications for similar scenarios.

## Case description

The patient, an 81-year-old female with a 25-year history of hypertension, presented with progressive dyspnea and fatigue over a 3-month period. Cardiac auscultation revealed a diastolic murmur over the left sternal border. Transthoracic echocardiography (TTE) demonstrated a tricuspid aortic valve with three symmetric cusps, without cusp thickening, prolapse, or calcification; it revealed a central AR jet (vena contracta: 7 mm), mild annular dilatation (27 mm), and significant aortic root dilatation (sinus of Valsalva: 48 mm). Additionally, TTE showed left ventricular enlargement (end-diastolic internal diameter/body surface area index: 35.5 mm/m^2^, end-systolic internal diameter/body surface area index: 25 mm/m^2^) (end-diastolic internal diameter/body surface area index: 35.5 mm/m^2^, end-systolic internal diameter/body surface area index: 25 mm/m^2^) with preserved left ventricular ejection fraction (60%). Given her high surgical risk, TAVR was planned.

**Figure 1:**
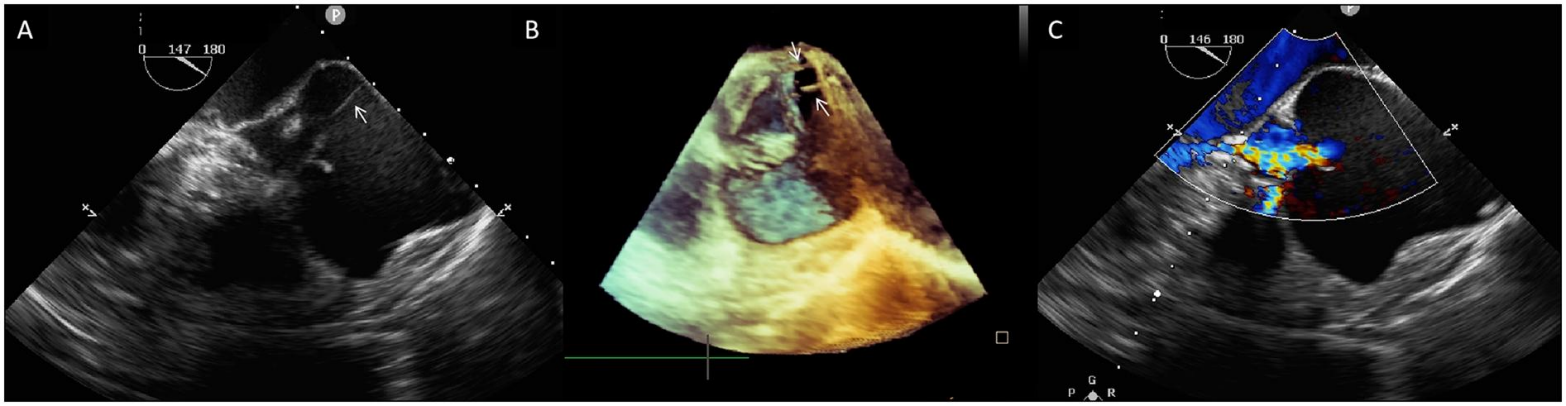
**(A)** Two-dimensional transesophageal echocardiography (TEE) shows a fibrous strand (arrow) extending from the aortic sinus wall to the free rim of the non-coronary aortic cusp. **(B)** Three-dimensional TEE shows two aortic chordae tendineae (white arrows)attaching the non-coronary cusp to the aortic sinus wall. **(C)** Color Doppler TEE demonstrates severe central aortic regurgitation (vena contracta: 7 mm).

Preoperatively, the patient underwent cardiac CT angiography for procedural planning and risk assessment, which demonstrated the small caliber of the bilateral femoral arteries; thus, transapical TAVR with the J-valve system (Genesis MedTech, China) was planned. Prosthetic size was determined by CT; however, cardiac CT failed to delineate detailed aortic root structures due to significant artifacts. Preoperative transesophageal echocardiography (TEE) was subsequently performed to characterize aortic root anatomy. Confirming TTE findings of the aortic root and cusps, TEE and 3D-TEE additionally identified two ACTs connecting the free margin of the non-coronary cusp to the aortic sinus wall (Figures 1A,B; Supplementary Videos S1, S2). Detailed assessment confirmed that AR was secondary to aortic sinus dilation, with no evidence of cusp restriction or excessive motion (Figure 1C, Supplementary Video S3). This assessment confirms aortic sinus dilation as the primary driver of AR, with ACTs not impacting native cusp motion.

This finding raised concerns that ACTs might compromise prosthetic valve stability or cause dislodgment during TAVR. However, anatomical assessment revealed ACTs were eccentrically positioned in the aortic sinus (deviating from the central axis), whereas graspers are deployed centrally in the sinus during TAVR. Thus, TAVR remained feasible, with precise intraoperative grasper positioning enabling ACTs avoidance.

**Figure 2:**
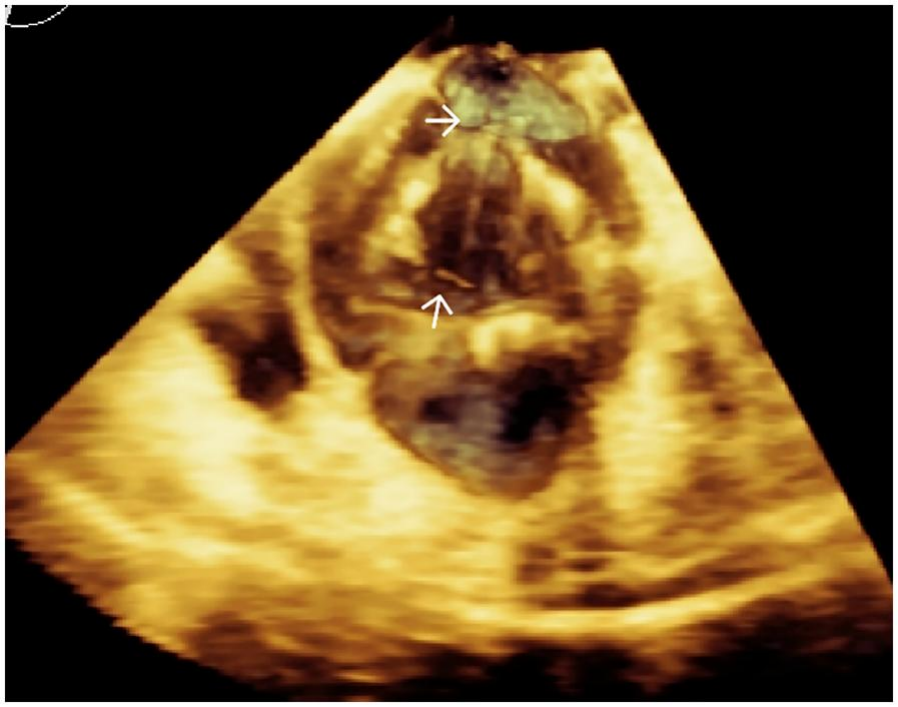
Three-dimensional TEE post-TAVR showing the self-expanding prosthetic valve with ruptured aortic chordae tendineae (white arrows) at the aortic root.

**Figure 3:**
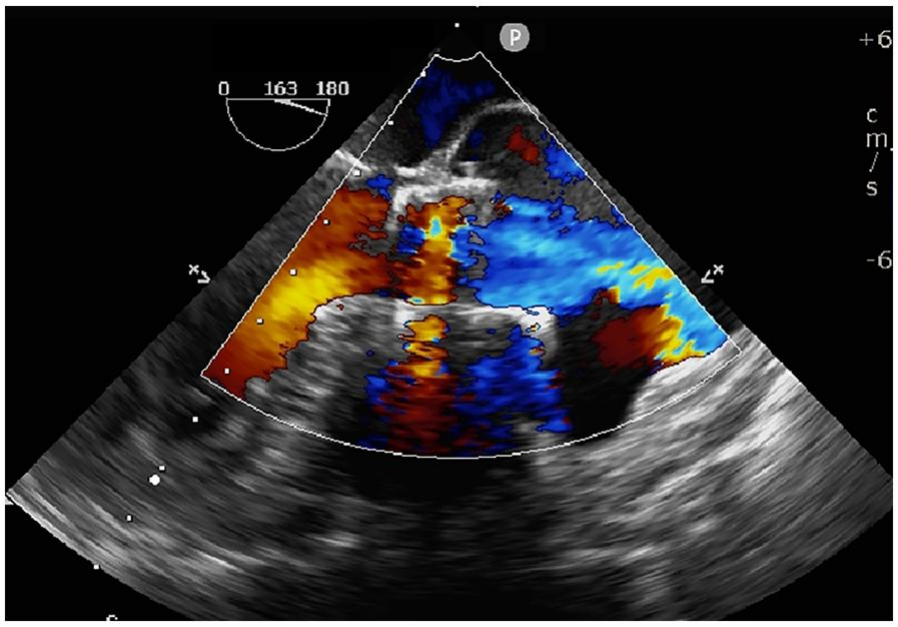
Intraoperative TEE confirming stable prosthetic valve position, absence of paravalvular leakage, and normal leaflet function.

Transapical access was achieved via a small intercostal incision over the left ventricular apex, using a dedicated delivery catheter and J-valve system. Under fluoroscopic guidance and TEE monitoring, three U-shaped nitinol graspers were deployed into the aortic sinuses, demonstrating stable morphology and positioning. A 29 mm self-expanding valve was then deployed and securely seated within the graspers. Intraoperative TEE confirmed optimal prosthetic position and stability, with no paravalvular regurgitation; notably, two ACTs ruptured, neither compromising valve stability (Figure 2; Supplementary Video S4). The TAVR valve was subsequently decoupled from the delivery system. Following delivery system retrieval, aortic root angiography and TEE confirmed absence of AR, paravalvular leakage, or coronary obstruction, with stable stent position and normal prosthetic function (Figure 3; Supplementary Video S5).

In the postoperative period, the patient recovered uneventfully without perioperative complications. At 6-month follow-up, she remained asymptomatic, and TTE confirmed optimal prosthetic position with no evidence of paravalvular leakage. Significant left ventricular reverse remodeling was observed; the left ventricular end-diastolic internal diameter/body surface area index decreased to 29.8 mm/m^2^, the end-systolic internal diameter/body surface area index decreased to 20.1 mm/m^2^, and left ventricular ejection fraction was 64%.

## Discussion

ACTs are rare embryonic remnants arising during early aortic valve development, with a hypothesized role in supporting cusps to maintain aortic valve coaptation (3). While prior reports link ACTs to severe AR or stenosis—predominantly in middle-aged to elderly individuals—*via* chordal rupture or cusp restriction, rare cases present ACT without valvular dysfunction, highlighting their variable clinical relevance (4–6). Their clinical implications remain poorly defined, particularly in TAVR.

We present a patient with AR secondary to aortic sinus dilation and incidentally detected ACTs; to our knowledge, this is the first report of transapical TAVR for AR with concomitant ACTs not directly causing valvular dysfunction. Unlike prior cases where ACT rupture drove AR, echocardiographic findings confirmed aortic sinus dilation as the primary driver of regurgitation, with ACTs showing no evidence of rupture or cusp motion restriction. This reinforces the hypothesis that most ACT remain clinically silent unless perturbed by age-related remodeling, functional imbalance, or spontaneous rupture, emphasizing the critical need to differentiate ACT as incidental anatomical variants from pathological contributors to valvular dysfunction.

ACTs may pose unique TAVR risks, including potential interference with valve deployment—risking grasper dislodgment or valve frame migration—necessitating strategies to mitigate these challenges. The successful outcome relied on three sequential technical strategies:

**Preprocedural detection**: TEE was pivotal for ACT identification, with superior sensitivity compared to TTE or cardiac CT (1). Three-dimensional TEE provided supplementary anatomical detail, confirming ACTs as eccentrically positioned fibrous strands attaching the non-coronary cusp to the aortic sinus wall, deviating from the central axis.

**Intraoperative grasper positioning**: The J-valve system's unique design—featuring three U-shaped nitinol graspers deployed centrally within the aortic sinuses—enabled avoidance of eccentrically located ACTs. This central deployment strategy is critical, as ACTs are typically attached to the aortic cusp (1), and their eccentric location minimizes overlap with grasper trajectories.

**Post-procedural validation**: Rupture of the ACTs during deployment was asymptomatic and did not impair valve function. This case provides the first clinical evidence that ACT rupture does not compromise prosthetic valve function or stability when optimal deployment techniques are employed, as confirmed by intraoperative TEE and 6-month follow-up echocardiographic data demonstrating stable valve position and absence of paravalvular leakage.

This case enhances understanding of ACT as incidental findings in AR and validates TAVR feasibility with appropriate imaging and intraoperative management. Clinically, it emphasizes that ACT presence does not preclude TAVR but requires meticulous intraoperative surveillance, reinforcing TEE's pivotal role in mitigating ACT-related procedural risks.

## Data Availability

The datasets presented in this article are not readily available because the patient data primarily consists of medical imaging images, which contain the patient's personal identifiable information (PII), rendering it inappropriate to upload and share. Requests to access the datasets should be directed to the corresponding author.
